# Fusing Diverse Decision Rules in 3D-Radiomics for Assisting Diagnosis of Lung Adenocarcinoma

**DOI:** 10.1007/s10278-024-00967-5

**Published:** 2024-04-02

**Authors:** He Ren, Qiubo Wang, Zhengguang Xiao, Runwei Mo, Jiachen Guo, Gareth Richard Hide, Mengting Tu, Yanan Zeng, Chen Ling, Ping Li

**Affiliations:** 1 Respiratory Department, Zhoupu Hospital, Shanghai University of Medicine and Health Sciences, Shanghai, China; 2 College of Medical Instrumentation and Collaborative Innovation Canter, Shanghai University of Medicine and Health Sciences, Shanghai, China; 3 Department of Radiology, Shanghai Tongren Hospital, Shanghai Jiao Tong University School of Medicine, Shanghai, China; 4 School of Mechanical and Power Engineering, East China University of Science and Technology, Shanghai, 200030 China; 5 Department of Surgery, Faculty of Health Sciences Medical School, University of the Witwatersrand, Parktown, Johannesburg, South Africa

**Keywords:** Diverse decision rules, 3D radiomics, Lung adenocarcinoma, Assisted diagnosis

## Abstract

This study aimed to develop an interpretable diagnostic model for subtyping of pulmonary adenocarcinoma, including minimally invasive adenocarcinoma (MIA), adenocarcinoma in situ (AIS), and invasive adenocarcinoma (IAC), by integrating 3D-radiomic features and clinical data. Data from multiple hospitals were collected, and 10 key features were selected from 1600 3D radiomic signatures and 11 radiological features. Diverse decision rules were extracted using ensemble learning methods (gradient boosting, random forest, and AdaBoost), fused, ranked, and selected via RuleFit and SHAP to construct a rule-based diagnostic model. The model’s performance was evaluated using AUC, precision, accuracy, recall, and *F*1-score and compared with other models. The rule-based diagnostic model exhibited excellent performance in the training, testing, and validation cohorts, with AUC values of 0.9621, 0.9529, and 0.8953, respectively. This model outperformed counterparts relying solely on selected features and previous research models. Specifically, the AUC values for the previous research models in the three cohorts were 0.851, 0.893, and 0.836. It is noteworthy that individual models employing GBDT, random forest, and AdaBoost demonstrated AUC values of 0.9391, 0.8681, and 0.9449 in the training cohort, 0.9093, 0.8722, and 0.9363 in the testing cohort, and 0.8440, 0.8640, and 0.8750 in the validation cohort, respectively. These results highlight the superiority of the rule-based diagnostic model in the assessment of lung adenocarcinoma subtypes, while also providing insights into the performance of individual models. Integrating diverse decision rules enhanced the accuracy and interpretability of the diagnostic model for lung adenocarcinoma subtypes. This approach bridges the gap between complex predictive models and clinical utility, offering valuable support to healthcare professionals and patients.

## Introduction

Lung cancer stands as a prominent contributor to both the incidence and mortality of malignant neoplasms on a global scale, occupying a significant position among malignancies [1, 2]. Among these, adenocarcinoma of the lung emerges as the most prevalent histological subtype. The incidence of lung adenocarcinoma has shown a gradual increase in recent years, particularly noticeable within the nonsmoker population [3–5]. The subtype classification of lung adenocarcinoma assumes paramount importance in the realms of treatment strategizing and prognostic assessment [6]. As delineated by the World Health Organization (WHO) classification, lung adenocarcinoma undergoes further subclassification into minimally invasive adenocarcinoma (MIA), adenocarcinoma in situ (AIS), and invasive adenocarcinoma (IAC) [7, 8]. MIA and AIS represent early-stage subtypes of lung adenocarcinoma, predominantly managed through surgical resection, which is associated with favorable prognosis [9, 10]. In contrast, addressing invasive adenocarcinoma (IAC) presents a more intricate clinical challenge. Treatment strategies for IAC typically encompass a comprehensive approach incorporating surgical resection, chemotherapy, and radiotherapy among other modalities [11]. The precision of subtype classification holds profound significance in guiding optimal therapeutic strategies and predicting patient prognoses [12–14]. In the realm of clinical medical informatics research, the utilization of CT imaging for the analysis and discernment of various subtypes of lung adenocarcinoma stands as a pivotal diagnostic modality [15–18].

Contemporary disease diagnostic research has witnessed remarkable accomplishments through the application of expansive data models, notably exemplified in the realm of adjunctive lung cancer diagnosis [19–21]. By amalgamating analysis of CT imagery with diverse patient multimodal data, a pronounced advancement in auxiliary diagnostic efficacy has been achieved [22–24]. However, in practical clinical use, these models face challenges in describing their internal decision-making processes and explaining internal features and predictive recommendations to medical professionals and patients. These issues have to some extent affected the trust that doctors place in the model predictions. Therefore, there is a need to explore interpretable methods and models to fulfill the requirements of clinical auxiliary diagnosis [25–27]. This emerging trend of enhancing model transparency and explicability is of paramount importance in bridging the gap between complex predictive models and their clinical utility. Chao utilized the RuleFit algorithm to explore valuable inflammatory rules for prognostic evaluation in nasopharyngeal carcinoma (NPC) patients. They identified 22 combined baseline hematological rules, achieving AUROCs of 0.69 and 0.64 in the training and validation cohorts, respectively. By developing risk-predictive rules from hematological indicators and clinical predictors, the final model demonstrated improved predictive precision over base models and exhibited strong generalizability [27]. Similarly, the study by Ke Wan addresses the challenge of estimating treatment effects based on real-world data in precision medicine. They propose an interpretable machine learning method using the RuleFit algorithm to estimate heterogeneous treatment effects (HTE). This approach improves interpretability while maintaining high prediction accuracy. The proposed method, applied to an HIV study dataset, demonstrates superior prediction accuracy compared to previous methods, offering a valuable tool for establishing interpretable models in precision medicine research [28].

In this study, a new diverse rule extraction method was proposed, and a new rule-based prognostic model was constructed to access the risk of lung adenocarcinoma subtypes. We amalgamated patient radiological features and radiomics features to select 10 key features. Employing the RuleFit method, diverse decision rules were generated by different ensemble learning methods including gradient boosting and random forest algorithms and AdaBoost. Then, 15 important decision rules are extracted. Subsequently, a rule-based auxiliary diagnostic model was constructed for assessing the risk of lung adenocarcinoma subtypes and conducted a comprehensive analysis.

## Methods and Materials

This study was conducted in accordance with the Declaration of Helsinki (as revised in 2013). The study was approved by the ethics committee of Shanghai University of Medicine & Health Sciences, and the written informed consent of patients was waived by the ethics committee because the study was a retrospective experiment and did not involve patient privacy.

### Patients

This study has utilized the data and initial data analysis methods from previous research. The dataset was collected from three hospitals (Shanghai Public Health Clinical Center, as hospital 1; Shanghai Ruijin Hospital, as hospital 2; Ningbo Beilun NO.2 Hospital, as hospital 3). Patients showed pulmonary nodules on chest CT scan and diagnosed as pulmonary adenocarcinomas based on pathologic analysis of surgical specimens that were selected for analyses. Other inclusion criteria were as follows: (1) routine CT examination had been conducted the month before surgery; (2) the maximum diameter of the tumor was less than 20 mm; (3) the preoperative CT layer thickness was less than 2 mm. Multiple nodules from the same patients were analyzed separately. The exclusion criteria were as follows: (1) obvious artifacts around the tumor were found on the CT image; (2) the contrast medium was used for CT examination. Meanwhile, the study also collected clinical information from patients (Table 1).

**Table 1:** Patient characteristic information in training cohort, testing cohort, and validation cohort

	**Training cohort (*n* = 282)**			**Testing cohort (*n* = 139)**			**Validation cohort (*n* = 85)**		
**Characteristics**	**MIA/AIS (*n* = 216)**	**IAC (*n* = 66)**	***P* value**	**MIA/AIS (*n* = 107)**	**IAC (*n* = 32)**	***P* value**	**MIA/AIS (*n* = 42)**	**IAC (*n* = 43)**	***P* value**
Age (years)	50.74 ± 11.19	54.60 ± 11.66	< 0.05	51.43 ± 11.31	53.28 ± 11.34	< 0.05	54.02 ± 13.39	62.74 ± 11.36	< 0.05
Gender			0.211						0.1148
Male	55	22		35	12		28	23	
Female	161	44		72	20		14	20	
Nodule type			< 0.05			< 0.05			< 0.05
PGGN	152	29		76	10		29	20	
MGGN	64	37		31	22		13	23	
Segment			0.7653			0.562			0.07993
Left upper lobe	62	18		34	7		14	7	
Left lower lobe	35	7		18	4		3	7	
Right upper lobe	73	26		33	15		11	21	
Right middle lobe	16	4		10	2		3	2	
Right lower lobe	30	11		12	4		11	6	
Average major axis (mm)	8.59 ± 2.31	11.02 ± 2.47	< 0.05	8.73 ± 2.55	11.19 ± 2.80	< 0.05	10.80 ± 4.26	18.58 ± 8.52	< 0.05
Average minor axis (mm)	7.15 ± 2.00	8.61 ± 1.70	< 0.05	7.31 ± 2.21	8.53 ± 1.91	< 0.05	8.67 ± 3.38	14.18 ± 5.95	< 0.05

### CT Image Acquisition

Unenhanced chest CT examinations encompassing entire lung scans were conducted on patients within hospital 1, hospital 2, and hospital 3. The CT images were acquired using distinct devices: a United-Imaging 760 CT device (tube voltage 120 kVp, tube current modulation 42–126 mA, reconstructed slice thickness 1.0 mm) and a Siemens Emotion 16 CT device (tube voltage 130 kVp, tube current modulation 34–123 mA, reconstructed slice thickness 1.0 mm) with 512 × 512 resolutions and 517.31 ± 16.11 exposure time at hospital 1; a Philips iCT 256 CT device (tube voltage 120 kVp, tube current modulation 161 mA, reconstructed slice thickness 1.0 mm) and a Philips Brilliance 16 CT device (tube voltage 120 kVp, tube current modulation 219 mA, reconstructed slice thickness 1.0 mm) for CT image acquisition with 512 × 512 resolution and 507.25 ± 8.79 exposure time in hospital 2 and hospital 3. To minimize artifacts due to respiratory motion, all patients underwent full inspiration during the chest CT examination.

### Features Extraction and Selection

Utilizing proprietary semi-automated software, all nodules in the selected CT images underwent segmentation [29]. Subsequent manual review by a radiologist with 6 years of experience, corroborated by another with 20 years of experience, ensured accurate segmentation. From the three-dimensional region of interest (ROI), a comprehensive set of 1600 3D radiomic signatures encompassing tumor attributes, histogram features, and high-order texture features were extracted. Open-source software (PyRadiomics 3.0.1) facilitated this extraction.

Radiographic features underwent independent assessment by the aforementioned experienced radiologists, with any disparities resolved through consensus. Noteworthy radiological features for each lesion encompassed margin clarity, lobulation presence, spiculation presence, pleural attachment (including pleural tag and indentation), air bronchogram presence, vessel changes, bubble lucency, nodule location, and major/minor axis measurements (computed from the ROI area using “PyRadiomics” software) [30].

**Fig. 1:**
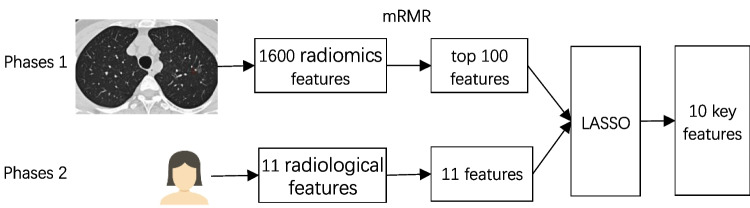
The process of the proposed 2-phases feature selection method. Phases 1: Utilizing PyRadiomics, 1600 imaging features were computed from lesion CT images. These features underwent analysis, and the mRMR method was applied to select the top 100 features. Phases 2: 11 radiological features from patient lesions were collected by professional radiologists. After merging these features with the previously obtained 100 features, the least absolute shrinkage and selection operator (LASSO) method was employed to extract the final set of 10 key features

A comprehensive set of 1611 features characterizing the 3D lesion area within CT images was assembled, encompassing 1600 radiomics signatures and 11 radiological features. The high dimension of radiomics signatures brought difficulties for modeling and analysis; therefore, a 2-phase feature selection approach was applied to remove redundancy and to identify the key features for identifying different subtypes of lung adenocarcinoma. The process of the proposed 2-phases feature selection approach is shown in Fig. 1.

**Fig. 2:**
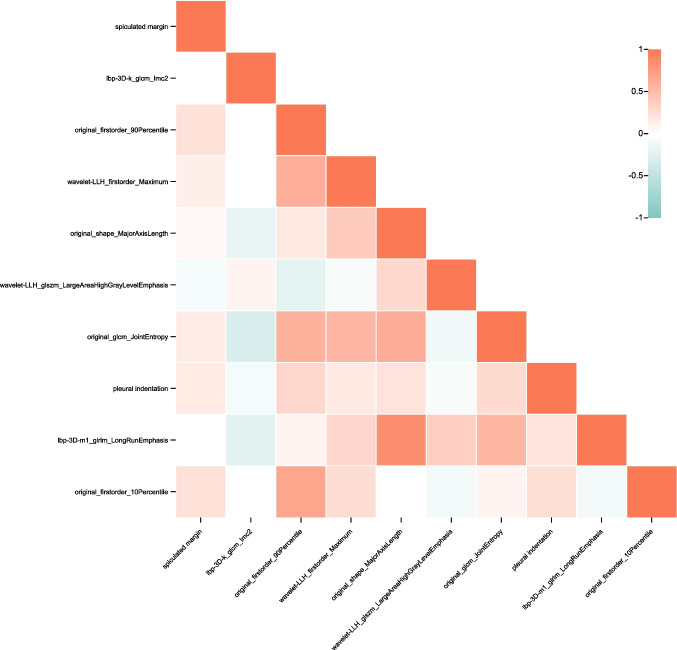
Correlation heatmap between the final 10 features

Initially, phase 1 is to reduce the redundancy in the radiomics signatures. All radiomics signatures underwent scaling normalization, followed by variance calculation to identify and subsequently exclude those nearing zero. Meanwhile, the pair-wise Spearman correlation analysis was used to calculate the correlation strength between features. Features whose absolute correlation value was above 0.9 were identified as strong interference features and were subsequently removed. After this preprocessing, the top 100 features were selected using the minimum redundancy maximum relevance (mRMR) method. Then, phase 2 is to identify key features related to the outcomes of lung cancer. The selected features in phase 1 were then employed as input for LASSO models to determine the optimal subsets for evaluating IAC and MIA/AIS. To account for potential randomness, the feature subset computation was iterated 500 times to ascertain the best result. Finally, 10 key features were sifted as the ultimate features, including “spiculated margin,” “lbp-3D-k_glcm_Imc2,” “original_firstorder_90Percentile,” “wavelet-LLH_firstorder_Maximum,” “original_shape_MajorAxisLength,” “wavelet-LLH_glszm_LargeAreaHighGrayLevelEmphasis,” “original_glcm_JointEntropy,” “pleural indentation,” “lbp-3D-m1_glrlm_LongRunEmphasis,” “original_firstorder_10Percentile” (Fig. 2).

### Generation of Rules

To obtain diverse rules related to different subtypes of lung adenocarcinoma, three ensemble learning methods were applied for extracting different types of decision rules, including gradient boosting decision trees (GBDT), random forest, and AdaBoosting. Firstly, we will explain how diverse rules are generated from ensemble learning methods.

Assume that $x$ represents the features of radiomics. The decision tree in each ensemble learning method is denoted as ${h}_{{\text{m}}}(x;{r}_{{\text{m}}})$. ${r}_{{\text{m}}}$ denotes the rules in the *m*th decision tree in an ensemble learning model. ${F}_{{\text{m}}}\left(X\right)$ denotes the ensemble learning model in the *m*th iteration, while $F\left(x\right)$ represents the integration of all meta-learners.

AdaBoosting is a boosting ensemble model, in which instances and meta-learners are reweighted in each iteration according to the prediction error achieved in the last iteration. The weighting mechanism can be expressed by Eq. (1):

1 $${F}_{{\text{m}}}\left(X\right)={F}_{{\text{m}}-1}\left(X\right)+{\beta }_{{\text{m}}}h\left(x;{r}_{{\text{m}}}\right)$$

2 $$F\left(x\right)={\text{sign}}\left({F}_{{\text{m}}}\left(x;{r}_{{\text{m}}}\right)\right)={\sum }_{{\text{m}}=1}^{{\text{M}}}{\beta }_{{\text{m}}}{F}_{{\text{m}}}\left(x;{r}_{{\text{m}}}\right)$$

where ${\beta }_{{\text{m}}}$ denotes the weight of the *m*th decision tree in an AdaBoosting. Therefore, the decision rules extracted from an AdaBoosting is weighted and sequentially correlated.

A GBDT is also a boosting ensemble model stacking the weak decision tree. Each tree is applied to predict the residuals of the preceding trees. In the *m*th iteration ($m=1:M$) of training, a GBDT is updated by summing the previous models and decision tree of the *m*th iteration multiplied by the weight ${\beta }_{{\text{m}}}$:

3 $$F\left(x\right)={F}_{{\text{m}}}\left(X\right)={F}_{{\text{m}}-1}\left(X\right)+{\beta }_{{\text{m}}}h\left(x;{r}_{{\text{m}}}\right)$$

Similar to AdaBoosting, the optimal step size ${\beta }_{{\text{m}}}$ is the weight of the current model, and ${r}_{{\text{m}}}$ denotes the rules of the *m*th decision tree in a GBDT model. Therefore, the decision rules extracted from a GBDT is also weighted and sequentially correlated.

Comparatively, a random forest is a collection of many independent decision trees. The prediction result of a random forest is determined by the average vote of these independent decision trees. The voting process can be formulated as:

4 $$F\left(X\right)={\text{sign}}\left({h}_{{\text{m}}}\left(x;{r}_{{\text{m}}}\right)\right)=\frac{1}{M}\sum\limits_{{\text{m}}=1}^{{\text{M}}}{h}_{{\text{m}}}\left(x;{r}_{{\text{m}}}\right)$$

The *M* in Eq. (4) denotes the number of trees in a random forest. Therefore, the decision rules extracted from a random forest are equal and independent, which are different from rules from GBDT or AdaBoosting.

To sum up, the decision rules extracted from GBDT and AdaBoosting are weighted and sequentially correlated, while decision rules extracted from the random forest are independent, determined by different architectures and mechanisms that decision trees integrated in these ensemble learning methods.

Three ensemble learning methods for rule generation are based on the selected 10 features. In the experimental phase, rule generation models were built using the GradientBoostingClassifier function (n_estimators = 500, max_depth = 5, learning_rate = 0.1), the RandomForestClassifier function (n_estimators = 500, min_samples_leaf = 1), and the AdaBoostClassifier function (base_estimator = DecisionTreeClassifier(max_depth = 5), n_estimators = 30, algorithm = ‘SAMME’) from the sklearn-ensemble package.

### RuleFit via Sparse Linear Method and Rule Selection

The RuleFit algorithm automatically extracted and selected decision rules generated from tree-based models such as random forest [31]. In the RuleFit, the original features, low-order and high-order rules, are combined as input to a sparse linear model, LASSO, which can sift the most useful rules through L1 penalty during the training process [32]. Compared with vanilla LASSO, the sparse linear model learned by RuleFit incorporates nonlinear feature interactions which can help us to capture more complex patterns hidden in high-dimensional 3D radiomics.

**Fig. 3:**
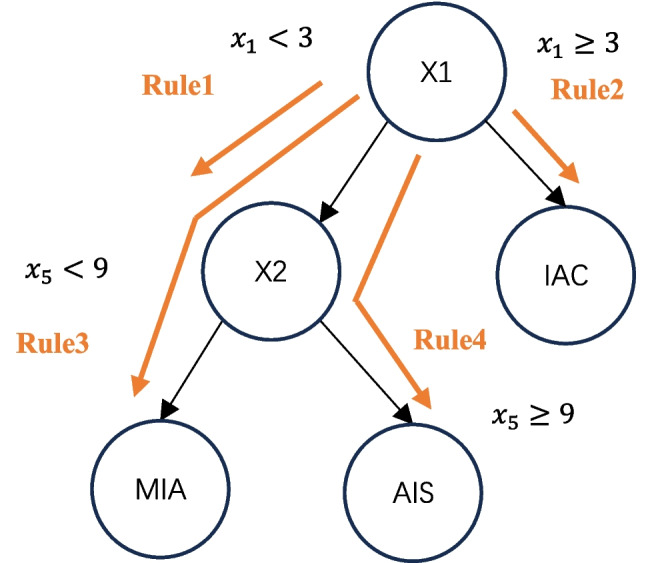
The process of radiomic rule extraction in a decision tree

In Fig. 3, each path through a tree can be transformed into a decision rule by combining the split decisions into a rule. Any model that generates decision trees can be used for RuleFit, such as random forest.

Diverse decision rules extracted from decision trees generated from three ensemble learning methods and the original radiomics features are combined as input for training a sparse linear model with LASSO, with the following structure:

5 $$\widehat{f}\left(x\right)={\widehat{\beta }}_{0}+\sum_{{\text{k}}=1}^{{\text{K}}}{\widehat{\alpha }}_{{\text{k}}}{r}_{{\text{k}}}\left(x\right)+\sum_{{\text{j}}=1}^{{\text{p}}}{\widehat{\beta }}_{{\text{j}}}{l}_{{\text{j}}}\left({x}_{{\text{j}}}\right)$$

where $\widehat{\alpha }$ is the estimated weight vector for the rule features and $\widehat{\beta }$ the weight vector for the original radiomics features. The loss function forces some of the weights to get a zero estimate due to the penalty item in LASSO:

6 $$\left({\left\{\widehat{\alpha }\right\}}_{1}^{{\text{K}}},{\left\{\widehat{\beta }\right\}}_{0}^{{\text{p}}}\right)={\text{arg}}\underset{{\left\{\widehat{\alpha }\right\}}_{1}^{{\text{K}}},{\left\{\widehat{\beta }\right\}}_{0}^{{\text{p}}}}{{\text{min}}}\sum\limits_{{\text{i}}=1}^{{\text{n}}}L\left({y}^{\left({\text{i}}\right)},f\left({x}^{\left({\text{i}}\right)}\right)\right)+\lambda \cdot \left(\sum\limits_{{\text{k}}=1}^{{\text{K}}}\left|{\alpha }_{{\text{k}}}\right|+\sum\limits_{{\text{j}}=1}^{{\text{p}}}\left|{b}_{{\text{j}}}\right|\right)$$

Diverse rules help to capture more important patterns hidden in the radiomics. The rule extraction, fusion, and selection process were automated using the RuleFit function (tree_generator = GradientBoostingClassifier/ RandomForestClassifier, rfmode = ‘classifier’, max_rules = 80), which extracted rules generated by the first two methods. A rule tree extraction method was designed to acquire rules generated by the AdaBoost method (GitHub code).

**Fig. 4:**
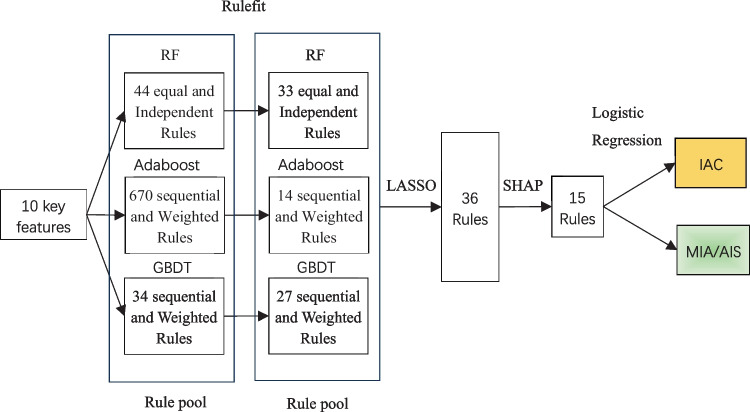
The process of diverse rules extraction, merge, selection, and model construction

To obtain a minimum number of rules and increase the explanation ability. Several steps are applied to reduce the scale of the rules related to different subtypes of lung adenocarcinoma. As demonstrated in Fig. 4, the rules are extracted and selected via RuleFit, LASSO, and Shapley additive explanations (SHAP).

For the rules extracted by the first two methods using RuleFit, those with an “importance” exceeding 0.1 were retained. Subsequently, the occurrence of each rule in various samples (true/false) was assessed and annotated with 0/1 labels. This process resulted in the creation of two novel dataset based on rules. Following the above approach, a new dataset based on rules was constructed for the rules generated using the AdaBoost method. Later, rules with a correlation to the diagnostic results exceeding 0.4 were retained. Merging the three rule-based datasets, followed by applying the LASSO method to select the optimal subset of rule-based features. Finally, employing the SHAP method, we conducted an analysis of the contributions of each rule within the initial model, retaining SHAP values not less than 0.5.

### Construction and Validation of Rule-Based Model

In this study, the diagnostic assistance model for IAC risk assessment was developed through multivariate logistic regression using the aforementioned selected rules. Subsequently, the model’s diagnostic performance was validated using both testing and validation datasets. ROC curves were generated, and the area under the curve (AUC) values were computed for the training, testing, and validation sets. Additionally, precision, accuracy, recall, and *F*1-score were calculated to comprehensively evaluate the model. Moreover, we conducted individual training and validation of the rules for the three methods using the same dataset. A comparative analysis was carried out against results from previous relevant research models to further evaluate the models’ performance. Furthermore, we also employed SHAP analysis to further investigate the rule features within the model.

### Statistical Analysis

Quantitative data were presented as either mean ± SD or median (25th–75th percentile), while qualitative data were represented as counts (*n*). Fisher’s exact test was used for intergroup comparisons of qualitative variables, and *t*-tests or Wilcoxon tests were employed for comparisons of quantitative variables between groups. A significance level of < 0.05 on a two-sided *P* value was considered statistically significant. The relevant statistical analyses in the study were conducted within the Python 3.9 environment. Additionally, certain graphical representations were generated using the features provided by the ChiPlot website (https://www.chiplot.online/).

## Results

### Patients

A total of 401 patients from hospital 1, meeting inclusion criteria, were identified with 421 pulmonary nodules (MIA/AIS 323, 76.7%; IAC 98, 23.3%). Simultaneously, hospital 2 and hospital 3 contributed 82 patients with 85 pulmonary nodules (MIA/AIS 42, 49.4%; IAC 43, 50.6%). Subsequently, this cohort of 483 patients, featuring 506 pulmonary nodules, was categorized into distinct groups: the training cohort encompassed 278 patients with 282 nodules spanning April 2015 to February 2017, the testing cohort included 123 patients with 139 nodules from February 2015 to December 2016, and the validation cohort comprised 82 patients with 85 nodules extending from September 2017 to February 2018. Importantly, no significant variations in gender and age were observed across these cohorts.

### Rule Generation, Selection, and Analysis

Feature selection was built upon previous research outcomes. Following dataset standardization, an analysis of feature variance and inter-feature correlations was conducted. Subsequently, the mRMR method was employed for feature ranking, followed by cross-validation for LASSO analysis on the remaining features. This process resulted in the identification of the top 10 optimal features.

Leveraging the aforementioned 10 features, the RuleFit method was employed with gradient boosting as the core to generate 34 rules (including 8 original features), while employing random forest as the core led to the creation of 44 rules (including 7 original features). Additionally, the AdaBoost method resulted in the formulation of 670 rules. Following the selection process, the gradient boosting approach retained 27 rules (including 6 original features), the random forest approach retained 33 rules (including 7 original features), and the AdaBoost method retained 14 rules. After applying the LASSO method, 36 rules were selected. Subsequently, utilizing SHAP analysis, 11 rules with low contribution values were removed.

**Fig. 5:**
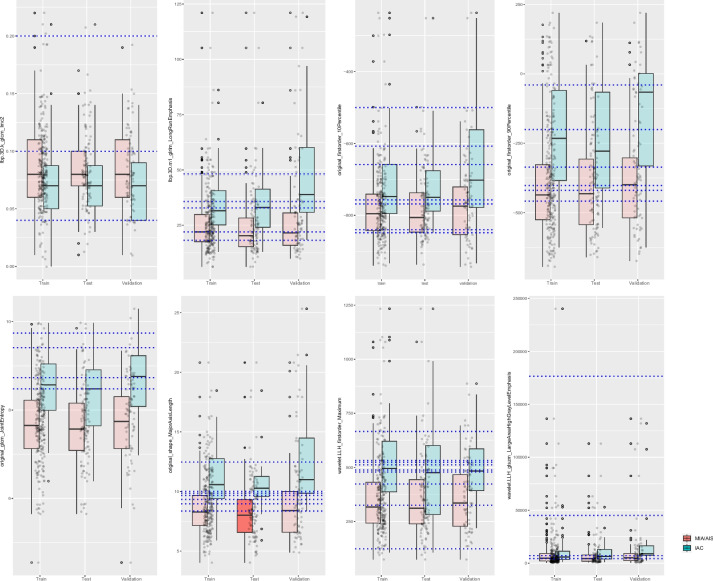
The distribution of continuous data features and decision values in rules. The blue dashed line represents the judgment value of the corresponding feature in the rule

Following further refinement, a total of 15 rules were retained, with 5 from gradient boosting, 2 from random forest, and 9 from AdaBoost (Table 2, use ID to represent the corresponding rule). Notably, there was 1 duplicated rule shared by gradient boosting and random forest. Furthermore, we will present the distribution of assessment metrics for each discrete feature in the range of feature values according to the defined rules. The results indicate that all features exhibit a certain degree of inter-group variation, notably the “orginal_shape_MajorAxisLength.” Additionally, a majority of the data points are concentrated within the overlapping region of the groups, which is also the range of particular interest for the assessment metrics. The main continuity numerical features and the distribution of judgment values in the rules are shown in Fig. 5.

**Table 2:** Information for 15 rules

**ID**	**Method**	**Rule**
Rule1	AdaBoost	original_shape_MajorAxisLength < = 12.46 & original_firstorder_10Percentile < = −768.75 & wavelet-LLH_glszm_LargeAreaHighGrayLevelEmphasis < = 45,043.45 & wavelet-LLH_firstorder_Maximum < = 487.20
Rule2	AdaBoost	original_glcm_JointEntropy < = 8.48 & original_shape_MajorAxisLength < = 10.01 & original_firstorder_10Percentile < = −658.70 & wavelet-LLH_firstorder_Maximum ∈ (122.71,525.63]
Rule3	AdaBoost	original_shape_MajorAxisLength < = 9.93 & original_firstorder_10Percentile < = −607.30 & original_glcm_JointEntropy < = 8.70 & wavelet-LLH_firstorder_Maximum ∈ (122.71,523.14]
Rule4	Gradient boosting	lbp-3D-k_glcm_Imc2 < = 0.20 & original_shape_MajorAxisLength < = 9.93 & original_firstorder_90Percentile < = −336.0
Rule5	Gradient boosting/random forest	spiculated margin
Rule6	AdaBoost	original_glcm_JointEntropy > 8.48 & original_firstorder_10Percentile > −848.55 & lbp-3D-k_glcm_Imc2 < = 0.10 & wavelet-LLH_firstorder_Maximum > 403.11
Rule7	AdaBoost	original_firstorder_90Percentile > −200.55 & lbp-3D-m1_glrlm_LongRunEmphasis > 18.23 & original_firstorder_10Percentile < = −499.90 & original_glcm_JointEntropy < = 9.74 & wavelet-LLH_firstorder_Maximum > 331.59
Rule8	Gradient boosting	gbc_wavelet-LLH_firstorder_Maximum < = 511.67 & wavelet-LLH_glszm_LargeAreaHighGrayLevelEmphasis < = 176,581.82 & lbp-3D-k_glcm_Imc2 > 0.04 & original_firstorder_90Percentile < = −405.40 & original_shape_MajorAxisLength > 9.93
Rule9	Gradient boosting	gbc_lbp-3D-m1_glrlm_LongRunEmphasis < = 48.18 & original_shape_MajorAxisLength > 8.84 & original_firstorder_90Percentile > −336.0 & original_glcm_JointEntropy < = 9.29 & wavelet-LLH_firstorder_Maximum > 478.69
Rule10	Gradient boosting	gbc_original_shape_MajorAxisLength > 8.33 & lbp-3D-m1_glrlm_LongRunEmphasis < = 32.81 & wavelet-LLH_firstorder_Maximum > 511.67
Rule11	AdaBoost	original_shape_MajorAxisLength > 8.96 & original_glcm_JointEntropy > 8.48 & original_firstorder_10Percentile > −839.90 & original_firstorder_90Percentile < = −39.80
Rule12	AdaBoost	original_shape_MajorAxisLength > 9.93 & lbp-3D-m1_glrlm_LongRunEmphasis ∈ (22.03, 35.69] & wavelet-LLH_glszm_LargeAreaHighGrayLevelEmphasis < = 8320.79 & original_firstorder_90Percentile > −458.35
Rule13	AdaBoost	original_shape_MajorAxisLength > 9.85 & lbp-3D-m1_glrlm_LongRunEmphasis ∈ (22.03,35.69] & original_firstorder_90Percentile > −419.95 & original_firstorder_10Percentile > −849.00
Rule14	AdaBoost	wavelet-LLH_glszm_LargeAreaHighGrayLevelEmphasis > 5460.49 & original_glcm_JointEntropy > 8.48 & wavelet-LLH_firstorder_Maximum < = 669.96 & original_firstorder_10Percentile > −839.90
Rule15	Random forest	rf_original_glcm_JointEntropy > 8.48 & pleural indentation = 1

**Fig. 6:**
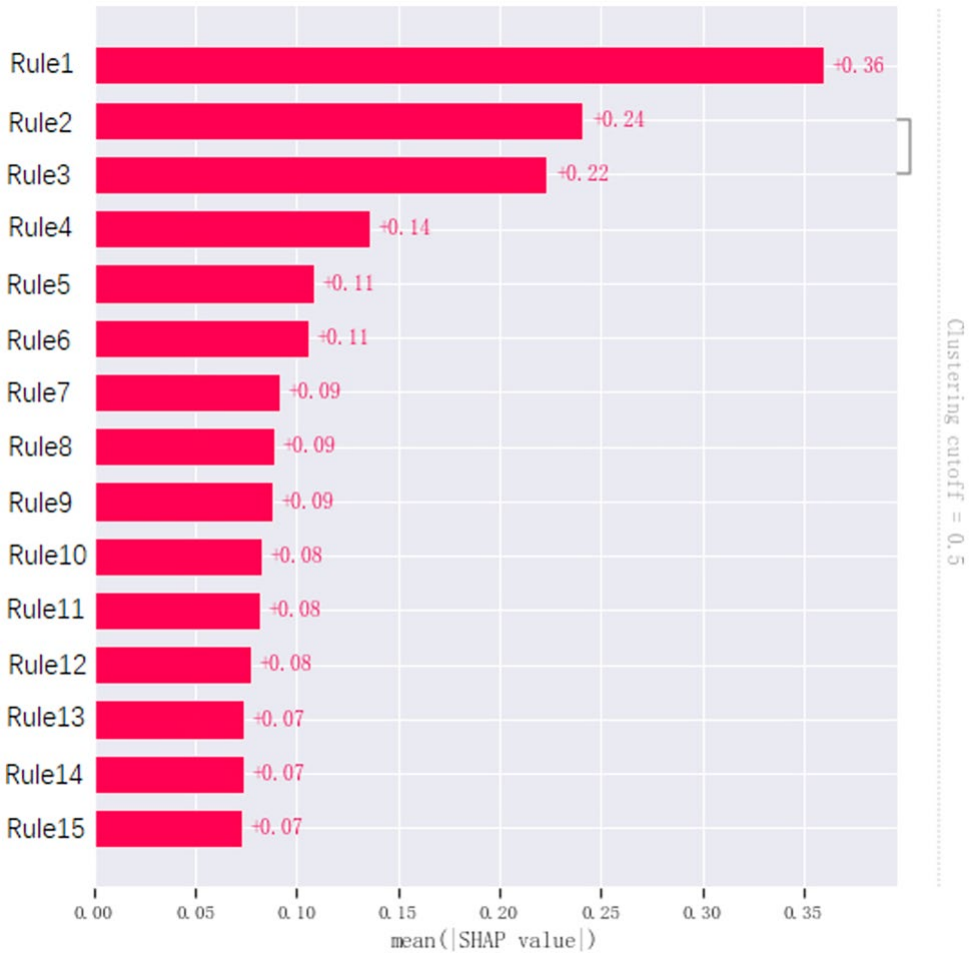
SHAP analysis of 15 rules

Additionally, we conducted SHAP to rank and scrutinize the individual features of the model. The results revealed that Rule1 exhibited significantly high feature importance within the model. Among the top 10 influential rules, 7 were generated by the AdaBoost method, 3 by the gradient boosting method, and 1 by the random forest method (which coincided with a rule generated by gradient boosting). Notably, “spiculated margin,” the only original features involved in model training, made a substantial contribution, ranking fourth in importance. Except for Rule 2 and Rule 3, there is no redundancy present in the remaining rules (Fig. 6).

### Model Construction, Evaluation, and Comparison

By conducting multivariate logistic regression analysis on the training dataset constructed from the aforementioned 15 rules, the formulated multivariate logistic regression equation is as follows.

$$\begin{aligned}Y=&-0.3265-0.7705 \times \mathrm{ Rule}1 - 0.4823 \times \mathrm{ Rule}2 - 0.4478 \times \mathrm{ Rule}3 - 0.2747 \\& \times \mathrm{ Rule}4 - 0.2537 \times \mathrm{ Rule}5 + 0.3795 \times \mathrm{ Rule}6 + 0.4433 \times \mathrm{ Rule}7- 0.3869 \\& \times \mathrm{ Rule}8 + 0.5280 \times \mathrm{ Rule}9 + 0.5978 \times \mathrm{ Rule}10+ 0.4615 \times \mathrm{ Rule}11+ 0.6856 \\& \times \mathrm{ Rule}12 + 0.4334 \times \mathrm{ Rule}13 + 0.5789 \times \mathrm{ Rule}14 + 0.3891 \times \mathrm{ Rule}15\end{aligned}$$

**Fig. 7:**
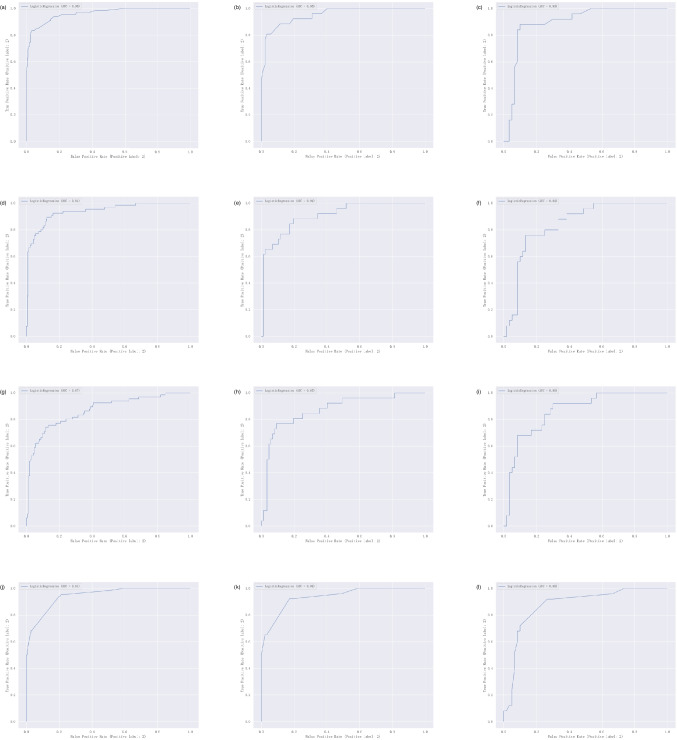
Performance of models based on diverse decision rules and respective models of three types of rules in the same dataset. **a**–**c** represents the performance of the mixed rule model in the training set, testing set, and validation set, respectively. **d**–**f** represents the performance of models based on gradient boosting rules in three datasets. **g**–**i** represents the performance of models based on random forest rules in three datasets. **j**–**l** represents the performance of the AdaBoost rule–based model in three datasets

The model demonstrates robust performance across all three datasets. In the training cohort, the model achieves an AUC of 0.9621 (95% CI, 0.9421–0.9822), an accuracy of 0.9433, a precision of 0.9464, a recall of 0.9815, and an *F*1 score of 0.9636. Similarly, in the testing cohort, the AUC is 0.9529 (95% CI, 0.9168–0.9889), accuracy is 0.9292, precision is 0.9341, recall is 0.9770, and *F*1 score is 0.9551. In the validation cohort, the AUC stands at 0.8953 (95% CI, 0.88297–0.9609), accuracy at 0.8706, precision at 0.9016, recall at 0.9167, and F1 score at 0.9091. The distinct rule-based feature subsets of the three methods exhibited individual AUC performances on the training, testing, and validation sets as follows: For gradient boosting, the values were 0.9391, 0.9093, and 0.8440 respectively. For random-forest, the values were 0.8681, 0.8722, and 0.8640 respectively. Lastly, for AdaBoost, the values were 0.9449, 0.9363, and 0.8750, respectively (Fig. 7 and Table 3).

**Table 3:** Performance of the models in datasets

**Models**	**Precision**	**Recall**	***F*1**	**Acc**
Diverse decision rules model in training set	0.9464	0.9815	0.9636	0.9433
Diverse decision rules model in testing set	0.9341	0.9771	0.9551	0.9291
Diverse decision rules model in validation set	0.918	0.9333	0.9256	0.8941

Moreover, in order to further assess the efficacy of the rules, we conducted comparisons between our model and other pertinent research models. We evaluated our model against one built using the selected 10 features on the same dataset [33]. Additionally, comparisons were made with the outcomes of three closely related studies [15, 34, 35]. The outcomes of these comparisons are summarized in Table 4.

**Table 4:** AUC performance of each model

**Models**	**Training set**	**Testing set**	**Validation set**
Diverse decision rules model	0.9621	0.9529	0.8953
Gradient boosting rules model	0.9391	0.9093	0.8440
Random forest rules model	0.8681	0.8722	0.8640
AdaBoost rules model	0.9449	0.9363	0.8750
10 features model with same dataset	0.8770	0.8930	0.8510
Paper15	0.8310	0.7920	0.8330
Paper32	0.885	0.808	-
Paper33	0.877	0.893	0.851

## Discussion

Extracting and fusing diverse decision rules help to capture more important patterns hidden in the radiomics, increasing both accuracy and interpretability for assessing the risk of lung adenocarcinoma subtypes. The exploration of diverse diagnostic rules has emerged as an urgent necessity in clinical decision support. Rule-based diagnostic assistance offers a visualizable and explainable diagnostic decision-making process, significantly enhancing decision-making and treatment guidance for both healthcare professionals and patients.

By analyzing 3D radiographic features and clinical characteristics, this study identified a crucial set of 10 features. Utilizing various algorithms for rule extraction and analysis, valuable rules were discovered, revealing relationships between data features and outcomes. Subsequently, a rule-based IAC risk assessment model was developed, offering visual and descriptive diagnostic insights. In comparison to models trained independently using the three rule-generation methods, the hybridized rule-based model exhibits superior accuracy. Compared to models constructed using the 10 selected features and those based on traditional features, it demonstrated notably enhanced accuracy and clinical significance.

In this study, RuleFit was utilized to learn a sparse linear model with the original radiomics features and diverse decision rules that capture key interactions between the original radiomics features. Three ensemble learning methods were applied to generate diverse decision rules from radiomics. Gradient boosting and AdaBoost generated weighted and sequentially correlated rules, while random-forest generated equal and independent rules. These diverse decision rules were fused, ranked, and selected through RuleFit and SHAP, which help us to construct a rule-based diagnostic model with both accuracy and interpretability. This research fills the gap of constructing simple and interpretable models and integrates nonlinear feature interactions in radiomics.

Among the final selection of 10 features, “original_shape_MajorAxisLength” represents the major axis length of the lesion, reflecting information about its size and elongation. “original_firstorder_90Percentile” and “original_firstorder_10Percentile” respectively indicate high and low CT value information within the lesion. “lbp-3D-k_glcm_Imc2” reflects relationships among adjacent pixel intensities, indicating texture heterogeneity within the lesion. “wavelet-LLH_firstorder_Maximum” reveals specific localized texture features. “wavelet-LLH_glszm_LargeAreaHighGrayLevelEmphasis” describes the presence of large areas with high CT values in the LLH subband after wavelet transformation, highlighting significant variations in CT value distribution. “original_glcm_JointEntropy” reflects the complexity of texture variations within the lesion. “lbp-3D-m1_glrlm_LongRunEmphasis” quantifies long-range trends of similar CT values within the lesion, emphasizing elongated structures or patterns present within it. Hence, the distribution of texture within the lesion area, notably the depiction of significant regions, stands as crucial discriminative information. Further complemented by key lesion features (spiculated margin and pleural indentation), this could offer more precise grounds for clinical decision support.

The top 5 high-contribution rules are highly representative:

- Rule 1: original_shape_MajorAxisLength <  = 12.46 & original_firstorder_10Percentile <  =  −768.75 &wavelet-LLH_glszm_LargeAreaHighGrayLevelEmphasis <  = 45,043.45&wavelet-LLH_firstorder_Maximum <  = 487.20. This rule was generated using the AdaBoost method. It first categorizes based on the lesion’s major axis length (as seen in Fig. 5, cases greater than 12.46 are mostly IAC); then considers the 10% CT value within the lesion (−768.75, relatively less common in IAC cases); next, analyzes regions with significant CT value variations (notably, 45,043.45 allows clear classification); and finally explores texture features in specific areas (cases with values greater than 487.20 are predominantly IAC).
- Rule 2: ada_original_glcm_JointEntropy <  = 8.48 & original_shape_MajorAxisLength <  = 10.01 & original_firstorder_10Percentile <  =  −658.70 & wavelet-LLH_firstorder_Maximum ∈ (122.71,525.63]. Also generated by the AdaBoost method, this rule first calculates the entropy reflecting the complexity of texture within the lesion area (with most values greater than 8.48 indicating IAC). Next, similar to Rule 1, it analyzes the lesion’s major axis length and the 10% CT value within the region. Finally, it examines regions with significant CT value variations (with the distinction being that this feature’s value is constrained within a specified range in the rule).
- Rule 3: original_shape_MajorAxisLength <  = 9.93 & original_firstorder_10Percentile <  = −607.30 & original_glcm_JointEntropy <  = 8.70 & wavelet-LLH_firstorder_Maximum ∈ (122.71, 523.14]. This rule was also generated by the AdaBoost method. It closely resembles Rule 2, with the key difference being the order of evaluation for “original_glcm_JointEntropy” and “original_shape_MajorAxisLength” in the rule (of course, the threshold values have also changed). This suggests that these two features possess similar discriminative capabilities. The four features in the rule may be crucial evaluation indicators for lesion recognition.
- Rule 4: lbp-3D-k_glcm_Imc2 <  = 0.20&original_shape_MajorAxisLength <  = 9.93& original_firstorder_90Percentile <  =  −336.0. This rule was generated by the gradient boosting method. It involves an analysis of the texture heterogeneity within the lesion, the lesion’s major axis length, and the 90th percentile CT value within the region.
- Rule 5: “spiculated margin.” This rule is highly unique; both the gradient boosting and random forest methods determined it to be valuable. Its sole original feature retained until the end and is a highly valuable lesion indicator.

Within the top five rules contributing most significantly to the model, “original_shape_MajorAxisLength” undoubtedly emerges as the pivotal discriminant. It holds two crucial thresholds, 12.46 and 9.93. From Fig. 5, it is evident that cases exceeding 12.46 are predominantly associated with IAC, while the range around 9.93 presents challenges in clear classification. Across all rules, this feature’s thresholds can be broadly categorized into three ranges (12.46, near 9.93, and near 8.5). The first and last ranges exhibit similar classification performance, while 9.93 resides in the region of most intricate classification. While we cannot solely rely on a single numerical value to determine the benign or malignant nature of a lesion, this particular discernment value seems to hold special significance, warranting further clinical validation. Another frequently employed feature is “wavelet-LLH_firstorder_Maximum.” Interestingly, this feature consistently serves as the final decisive element in the rules. As a descriptor of specific localized texture characteristics, it complements the rules effectively yet appears to lack robust classification capability. Among various features representing lesion texture, “original_glcm_JointEntropy” is the most frequently utilized in the rules. This feature reflects entropy, indicating the randomness and disorder in pixel distribution, effectively capturing diverse lesion manifestations on CT scans (such as ground-glass opacities, vascular cluster signs).

“Spiculated margin” and “pleural indentation,” as the preserved lesion indicator features, appear less active in the rule set. “Spiculated margin,” despite being a significant sign of lesion activity, has made noteworthy contributions to the model. However, apart from its presence in Rule 5 in its original form, it is absent in other rules. Notably, “pleural indentation” is only used once, in Rule 15. This could be attributed to the strong discriminatory power inherent in “spiculated margin.” Moreover, the comprehensive evaluation provided by various features reflecting lesion texture, shape, and intensity distribution already effectively captures the manifestation of lesion characteristics.

Upon comparing all the rules, it is evident that a majority of them typically follow a decision sequence of first assessing morphological characteristics (such as “original_shape_MajorAxisLength”), then scrutinizing key CT values (like “original_firstorder_10Percentile”), and finally analyzing texture (such as “original_glcm_JointEntropy”). This decision logic closely parallels the routine image interpretation approach adopted by radiologists.

This study also had some limitations. Despite our efforts to include multiple data sources, the sample size remains relatively small. Additionally, all samples were collected based on real clinical cases, without considering the balance between IAC and MIA/AIS groups and consistency in CT equipment parameters. These factors might introduce certain interference in the rules due to data constraints. In future research, we intend to expand the sample size and distribution to validate the model’s performance. Furthermore, the 3D radiomics features used in the study were calculated using a single software package, potentially leading to bias. This underscores the need for comprehensive collection and computation of high-dimensional image features in subsequent studies to discover more effective feature rules.

## Conclusions

This study integrated 3D radiomic features and clinical data and constructed a rule-based diagnostic model for assessing the risk of lung adenocarcinoma subtypes. Diverse rules were extracted through three ensemble-learning algorithms and then ranked and selected via RuleFit and SHAP. Ultimately, an IAC risk assessment model based on 15 rules was constructed. This model demonstrated significantly improved performance compared to similar feature-based models. Through analysis, several key features and important decision rules with decision-making value were identified.

## Data Availability

The corresponding author can provide the datasets used and/or analyzed during the current study upon reasonable request.

## Abbreviations

MIA: Minimally invasive adenocarcinoma
AIS: Adenocarcinoma in situ
IAC: Invasive adenocarcinoma
ROI: Region of interest
LASSO: Least absolute shrinkage and selection operator
GBDT: Gradient boosting decision trees
SHAP: Shapley additive explanations
